# Triclinic modification of tetra­kis­(triethyl­ammonium) dihydrogeno­deca­vanadate(V)

**DOI:** 10.1107/S1600536811019696

**Published:** 2011-05-28

**Authors:** Seik Weng Ng

**Affiliations:** a Department of Chemistry, University of Malaya, 50603 Kuala Lumpur, Malaysia

## Abstract

In the title ammonium polyoxometallate salt, (C_6_H_16_N)_4_[H_2_V_10_O_28_], the anion features O atoms engaged in μ_6_-, μ_3_- and μ_2_-bridging of adjacent V^V^ atoms, confering an octa­hedral coordination at each of the twenty unique metal atoms. Two anions are linked by μ_3_- and μ_2_-bridged OH units across a center of inversion, forming a dimer which is linked to the cations by N—H⋯O hydrogen bonds. The cation is disordered over two positions in a 0.776 (4):0.224 (4) ratio in one of the two independent ion pairs in the asymmetric unit, and 0.627 (10):0.373 (10) in the other.

## Related literature

For the monoclinic modification, see: Correia *et al.* (2004 ▶); Sarkar & Pal (2008 ▶).
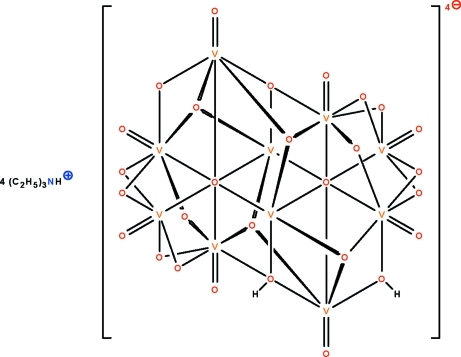


## Experimental

### 

#### Crystal data

(C_6_H_16_N)_4_[H_2_V_10_O_28_]
*M* *_r_* = 1368.21
Triclinic, 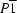

*a* = 13.1735 (4) Å
*b* = 20.0061 (7) Å
*c* = 20.2427 (7) Å
α = 62.669 (3)°
β = 86.263 (3)°
γ = 76.979 (3)°
*V* = 4613.0 (3) Å^3^
*Z* = 4
Mo *K*α radiation
μ = 2.02 mm^−1^
*T* = 100 K
0.20 × 0.15 × 0.10 mm

#### Data collection

Agilent SuperNova Dual diffractometer with an Atlas detector
Absorption correction: multi-scan (*CrysAlis PRO*; Agilent, 2010 ▶) *T*_min_ = 0.688, *T*_max_ = 0.824
38533 measured reflections
20465 independent reflections
13087 reflections with *I* > 2σ(*I*)
*R*_int_ = 0.041

#### Refinement

*R*[*F* ^2^ > 2σ(*F* ^2^)] = 0.049
*wR*(*F* ^2^) = 0.133
*S* = 1.04
20465 reflections
1230 parameters
132 restraints
H-atom parameters constrained
Δρ_max_ = 1.47 e Å^−3^
Δρ_min_ = −0.79 e Å^−3^

### 

Data collection: *CrysAlis PRO* (Agilent, 2010 ▶); cell refinement: *CrysAlis PRO*; data reduction: *CrysAlis PRO*; program(s) used to solve structure: *SHELXS97* (Sheldrick, 2008 ▶); program(s) used to refine structure: *SHELXL97* (Sheldrick, 2008 ▶); molecular graphics: *X-SEED* (Barbour, 2001 ▶); software used to prepare material for publication: *publCIF* (Westrip, 2010 ▶).

## Supplementary Material

Crystal structure: contains datablocks global, I. DOI: 10.1107/S1600536811019696/zs2113sup1.cif

Structure factors: contains datablocks I. DOI: 10.1107/S1600536811019696/zs2113Isup2.hkl

Additional supplementary materials:  crystallographic information; 3D view; checkCIF report

## Figures and Tables

**Table 1:** Hydrogen-bond geometry (Å, °)

*D*—H⋯*A*	*D*—H	H⋯*A*	*D*⋯*A*	*D*—H⋯*A*
N1—H1⋯O3	0.88	2.06	2.937 (4)	175
N2—H2⋯O14	0.88	1.89	2.765 (4)	176
N3—H3⋯O41	0.88	1.82	2.699 (4)	175
N4—H4⋯O9	0.88	2.13	2.968 (9)	160
N4′—H4′⋯O9	0.88	1.99	2.830 (16)	158
N5—H5⋯O26	0.88	2.39	2.981 (5)	125
N5—H5⋯O28	0.88	2.38	3.187 (5)	153
N6—H6⋯O51	0.88	1.96	2.823 (4)	167
N7—H7⋯O34	0.88	2.33	3.061 (4)	141
N7—H7⋯O45	0.88	2.44	3.156 (5)	139
N7—H7⋯O46	0.88	2.40	3.139 (4)	142
N8—H8⋯O52	0.88	1.91	2.766 (4)	162
O15—H15⋯O18^i^	0.84	1.99	2.819 (4)	171
O17—H17⋯O16^i^	0.84	1.90	2.715 (4)	162
O54—H54⋯O55^ii^	0.84	1.88	2.686 (4)	159
O56—H56⋯O53^ii^	0.84	1.94	2.775 (4)	177

Symmetry codes: (i) 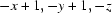; (ii) 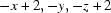.

**Fig. 1.**
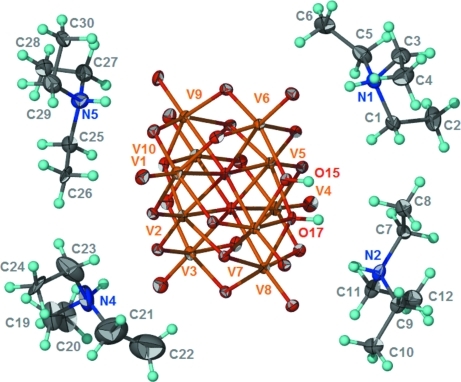
Thermal ellipsoid plot (Barbour, 2001) of one independent 4(C2H5)3NH+ [H2O28V10]4- ion-pair at the 70% probability level. Hydrogen atoms are drawn as spheres of arbitrary radius. The disorder is not shown and O atoms other than those involved in hydrogen bonding are not labeled.

**Fig. 2.**
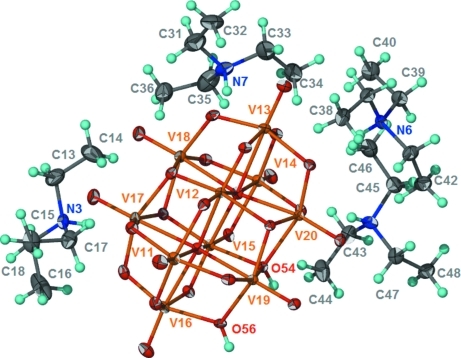
Thermal ellipsoid plot (Barbour, 2001) of the second independent 4(C2H5)3NH+ [H2O28V10]4- ion-pair at the 70% probability level. Hydrogen atoms are drawn as spheres of arbitrary radius. The disorder is not shown and O atoms other than those involved in hydrogen bonding are not labeled.

**Fig. 3.**
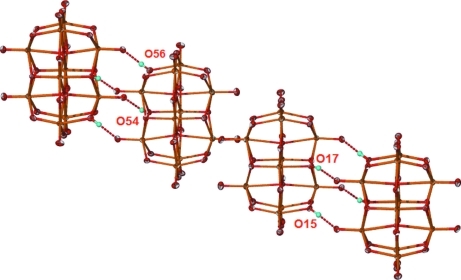
Hydrogen bonding linking the two independent [H2O28V10]4- anions into a centrosymmetric dimer.

## supplementary crystallographic information

### Comment

The polyoxometallate, 4(C_2_H_5_)_3_NH^+^ [H_2_O_28_V_10_]^4-^, tetrakis(triethylammonium) bis(µ_6_-oxo)-(µ_3_-hydroxo)-tris(µ_3_-oxo)-(µ_2_-hydroxo)-trideca(µ_2_-oxo)-octaoxo-decavanadate(V) (Scheme I) was the product obtained by reacting triethylammonium *D*,*L*-aminopropionate, salicylaldehyde and vanadyl(IV) sulfate in methanol (Correia *et al.*, 2004). The POM can also be synthesized from the reaction of triethylamine and vanadium(V) oxide in water (Sarkar & Pal, 2008). The compound, which belongs to the monoclinic *P*2_1_/*c* space group, features the dihydrogenodecavanadate tetraanion surrounded by the cations; two tetraanions are linked by a pair of O–H···O hydrogen bonds to generate a dimer. In the present triclinic modification, the ion similarly features oxygen atoms engaged in µ_6_–, µ_3_– and µ_2_-bridging of adjacent vanadium(V) atoms to confer octahedral coordination at the metal centers (Figs. 1 & 2). Two ions are linked by µ_3_-bridged and µ_2_-bridged OH units across a center-of-inversion to form a dimer (Fig. 3). The dimers are linked to the cations by N–H···O hydrogen bonds (Table 1).

### Experimental

Vanadyl(IV) sulfate (0.25 g, 1 mmol), 4-oxo-1,4-pyran-2,6-dicarboxylic acid (chelidonic acid) (0.18 g, 1 mmol) and triethylamine (0.4 ml, 3 mmol) were heated in water (100 ml) for an hour. The solution was filtered and upon slow evaporation of the filtrate afforded orange prisms of the title compound as a side product in low yield.

### Refinement

Carbon-bound H-atoms were placed in calculated positions [C—H = 0.95–0.98 Å, N—H = 0.88 Å; *U*_iso_(H) 1.2 to 1.5*U*_eq_(C)] and were included in the refinement in the riding model approximation. For each tetraanion, only two of the twenty-eight O atoms were within the range for hydrogen-bonding interaction with the O atom of an adjacent anion. Of the two, one is connected to two V atoms and the other to three V atoms. Hydrogen atoms were placed in calculated positions for these two and their displacement parameters were tied by a factor of 1.5. Two of the eight cations are disordered. The occupancies refined to 0.627 (1) and 0.776 (1) under these conditions: the carbon–carbon and carbon–nitrogen distances were restrained to 1.50±0.01 Å; the distances of the 1,3-related atoms were restrained to 2.35±0.01 Å. The atoms of the minor components were refined isotropically with the thermal parameters of the primed atoms set to those of the unprimed ones. The displacement parameters of the unprimed (major component) atoms were refined anisotropically but they were tightly restrained. The final difference Fourier map had a peak in the vicinity of the cation with the N4/N4' atoms. The (4 8 4) reflection was omitted owing to bad disagreement between observed and calculated structure factors.

### Crystal data

**Table:**

(C_6_H_16_N)_4_[H_2_V_10_O_28_]	*Z* = 4
*M**_r_* = 1368.21	*F*(000) = 2768
Triclinic, *P*1	*D*_x_ = 1.970 Mg m^−^^3^
Hall symbol: -P 1	Mo *K*α radiation, λ = 0.71073 Å
*a* = 13.1735 (4) Å	Cell parameters from 9283 reflections
*b* = 20.0061 (7) Å	θ = 2.3–29.4°
*c* = 20.2427 (7) Å	µ = 2.02 mm^−^^1^
α = 62.669 (3)°	*T* = 100 K
β = 86.263 (3)°	Block, orange
γ = 76.979 (3)°	0.20 × 0.15 × 0.10 mm
*V* = 4613.0 (3) Å^3^	

### Data collection

**Table:**

Agilent SuperNova Dual diffractometer with an Atlas detector	20465 independent reflections
Radiation source: SuperNova (Mo) X-ray Source	13087 reflections with *I* > 2σ(*I*)
Mirror	*R*_int_ = 0.041
Detector resolution: 10.4041 pixels mm^-1^	θ_max_ = 27.5°, θ_min_ = 2.3°
ω scans	*h* = −17→15
Absorption correction: multi-scan (*CrysAlis PRO*; Agilent, 2010)	*k* = −25→25
*T*_min_ = 0.688, *T*_max_ = 0.824	*l* = −25→20
38533 measured reflections	

### Refinement

**Table:**

Refinement on *F*^2^	Primary atom site location: structure-invariant direct methods
Least-squares matrix: full	Secondary atom site location: difference Fourier map
*R*[*F*^2^ > 2σ(*F*^2^)] = 0.049	Hydrogen site location: inferred from neighbouring sites
*wR*(*F*^2^) = 0.133	H-atom parameters constrained
*S* = 1.04	*w* = 1/[σ^2^(*F*_o_^2^) + (0.0374*P*)^2^ + 4.321*P*] where *P* = (*F*_o_^2^ + 2*F*_c_^2^)/3
20465 reflections	(Δ/σ)_max_ = 0.001
1230 parameters	Δρ_max_ = 1.47 e Å^−^^3^
132 restraints	Δρ_min_ = −0.79 e Å^−^^3^

### Fractional atomic coordinates and isotropic or equivalent isotropic displacement parameters (Å^2^)

**Table:**

	*x*	*y*	*z*	*U*_iso_*/*U*_eq_	Occ. (<1)
V1	0.77694 (5)	0.48056 (4)	0.19071 (4)	0.01186 (15)	
V2	0.64243 (5)	0.35842 (4)	0.26737 (4)	0.00949 (15)	
V3	0.49939 (5)	0.24453 (4)	0.34541 (4)	0.01232 (15)	
V4	0.30463 (5)	0.37271 (4)	0.26944 (4)	0.01214 (15)	
V5	0.43466 (5)	0.49681 (4)	0.19648 (4)	0.00913 (15)	
V6	0.57976 (5)	0.60703 (4)	0.12594 (4)	0.01115 (15)	
V7	0.62844 (5)	0.45585 (4)	0.09648 (4)	0.00919 (15)	
V8	0.48033 (5)	0.33937 (4)	0.17231 (4)	0.00959 (15)	
V9	0.60521 (5)	0.50398 (4)	0.29472 (4)	0.01161 (15)	
V10	0.45934 (5)	0.39236 (4)	0.36584 (4)	0.01140 (15)	
V11	0.71402 (5)	0.02601 (4)	0.81411 (4)	0.01270 (15)	
V12	0.84886 (5)	0.14660 (4)	0.73596 (4)	0.00935 (15)	
V13	0.99257 (5)	0.25928 (4)	0.65730 (4)	0.01173 (15)	
V14	1.18718 (5)	0.12853 (4)	0.72405 (4)	0.01212 (15)	
V15	1.05599 (5)	0.00684 (4)	0.79868 (4)	0.00968 (15)	
V16	0.91101 (5)	−0.10410 (4)	0.87182 (4)	0.01230 (15)	
V17	0.87818 (5)	0.00486 (4)	0.70336 (4)	0.01267 (15)	
V18	1.02116 (5)	0.11650 (4)	0.62872 (4)	0.01241 (15)	
V19	0.87140 (5)	0.04489 (4)	0.90605 (4)	0.00972 (15)	
V20	1.02180 (5)	0.15933 (4)	0.82893 (4)	0.00976 (15)	
O1	0.9002 (2)	0.47302 (16)	0.19015 (15)	0.0167 (6)	
O2	0.7239 (2)	0.58138 (15)	0.13065 (14)	0.0134 (6)	
O3	0.5482 (2)	0.69759 (15)	0.06765 (14)	0.0145 (6)	
O4	0.4329 (2)	0.59004 (15)	0.13614 (14)	0.0124 (6)	
O5	0.3104 (2)	0.48724 (15)	0.20236 (14)	0.0130 (6)	
O6	0.1800 (2)	0.38170 (17)	0.26912 (15)	0.0169 (6)	
O7	0.3571 (2)	0.27151 (16)	0.33046 (14)	0.0139 (6)	
O8	0.5246 (2)	0.15373 (16)	0.40010 (15)	0.0182 (7)	
O9	0.6476 (2)	0.26382 (15)	0.32380 (14)	0.0121 (6)	
O10	0.7667 (2)	0.36935 (15)	0.25483 (14)	0.0121 (6)	
O11	0.5996 (2)	0.47965 (15)	0.19271 (14)	0.0101 (6)	
O12	0.4799 (2)	0.37827 (15)	0.25905 (14)	0.0095 (6)	
O13	0.7568 (2)	0.45384 (15)	0.11218 (14)	0.0110 (6)	
O14	0.3377 (2)	0.36151 (15)	0.18450 (14)	0.0117 (6)	
O15	0.5855 (2)	0.56775 (15)	0.05089 (14)	0.0112 (6)	
H15	0.5712	0.5961	0.0052	0.017*	
O16	0.6266 (2)	0.44679 (15)	0.02124 (14)	0.0125 (6)	
O17	0.4674 (2)	0.46055 (15)	0.11595 (14)	0.0093 (6)	
H17	0.4272	0.4847	0.0778	0.014*	
O18	0.4837 (2)	0.32842 (15)	0.09755 (14)	0.0135 (6)	
O19	0.5097 (2)	0.24799 (15)	0.24725 (14)	0.0115 (6)	
O20	0.6233 (2)	0.35625 (15)	0.17384 (14)	0.0106 (6)	
O21	0.7416 (2)	0.49103 (16)	0.27595 (14)	0.0140 (6)	
O22	0.5725 (2)	0.60376 (15)	0.21513 (14)	0.0123 (6)	
O23	0.4558 (2)	0.49871 (15)	0.28460 (14)	0.0115 (6)	
O24	0.3275 (2)	0.39811 (16)	0.34592 (14)	0.0142 (6)	
O25	0.4972 (2)	0.28613 (15)	0.40824 (14)	0.0140 (6)	
O26	0.4561 (2)	0.40915 (16)	0.43642 (14)	0.0158 (6)	
O27	0.6107 (2)	0.39441 (15)	0.33944 (14)	0.0119 (6)	
O28	0.6005 (2)	0.51805 (16)	0.36690 (15)	0.0165 (6)	
O29	0.7669 (2)	−0.07452 (15)	0.86913 (15)	0.0137 (6)	
O30	0.5907 (2)	0.03358 (16)	0.81715 (15)	0.0178 (6)	
O31	0.7244 (2)	0.13831 (15)	0.75029 (14)	0.0131 (6)	
O32	0.9684 (2)	0.35063 (16)	0.60587 (15)	0.0166 (6)	
O33	0.8472 (2)	0.24138 (15)	0.68300 (14)	0.0120 (6)	
O34	1.1353 (2)	0.23117 (15)	0.66626 (14)	0.0127 (6)	
O35	1.3102 (2)	0.12123 (16)	0.71899 (15)	0.0164 (6)	
O36	1.1812 (2)	0.01466 (15)	0.78935 (15)	0.0130 (6)	
O37	1.0575 (2)	−0.08693 (15)	0.85708 (14)	0.0121 (6)	
O38	0.9343 (2)	−0.19435 (16)	0.92642 (16)	0.0185 (7)	
O39	0.8912 (2)	0.02654 (15)	0.80575 (14)	0.0107 (6)	
O40	1.0128 (2)	0.12615 (15)	0.73946 (14)	0.0110 (6)	
O41	0.7417 (2)	0.01906 (16)	0.72553 (15)	0.0139 (6)	
O42	0.8759 (2)	−0.00794 (17)	0.63138 (15)	0.0191 (7)	
O43	0.8729 (2)	0.11523 (15)	0.65973 (14)	0.0124 (6)	
O44	1.0201 (2)	0.10541 (16)	0.55509 (15)	0.0176 (6)	
O45	0.9883 (2)	0.22217 (16)	0.59116 (14)	0.0138 (6)	
O46	1.1563 (2)	0.10872 (16)	0.64621 (14)	0.0138 (6)	
O47	1.0280 (2)	0.00889 (15)	0.70875 (14)	0.0115 (6)	
O48	0.9115 (2)	−0.09615 (16)	0.77991 (15)	0.0142 (6)	
O49	0.7417 (2)	0.04857 (15)	0.89374 (14)	0.0131 (6)	
O50	0.8769 (2)	0.14513 (15)	0.83057 (14)	0.0097 (6)	
O51	0.9921 (2)	0.25453 (15)	0.75635 (14)	0.0120 (6)	
O52	1.1606 (2)	0.13761 (15)	0.81181 (14)	0.0119 (6)	
O53	1.0255 (2)	0.16673 (15)	0.90495 (14)	0.0133 (6)	
O54	1.0318 (2)	0.03896 (15)	0.88213 (14)	0.0098 (6)	
H54	1.0739	0.0132	0.9191	0.015*	
O55	0.8771 (2)	0.05317 (15)	0.98159 (14)	0.0127 (6)	
O56	0.9129 (2)	−0.06647 (15)	0.94731 (14)	0.0128 (6)	
H56	0.9299	−0.0959	0.9925	0.019*	
N1	0.3270 (3)	0.7559 (2)	0.01839 (19)	0.0174 (8)	
H1	0.3935	0.7366	0.0318	0.021*	
N2	0.1601 (3)	0.3797 (2)	0.10687 (18)	0.0172 (8)	
H2	0.2182	0.3734	0.1302	0.021*	
N3	0.5922 (3)	−0.01294 (19)	0.66663 (19)	0.0173 (8)	
H3	0.6384	−0.0025	0.6879	0.021*	
N4	0.8593 (7)	0.1626 (4)	0.3509 (4)	0.041 (2)	0.627 (10)
H4	0.7948	0.1849	0.3545	0.050*	0.627 (10)
N4'	0.8484 (12)	0.1702 (7)	0.3354 (6)	0.041*	0.373 (10)
H4'	0.7837	0.1889	0.3423	0.050*	0.373 (10)
N5	0.6502 (3)	0.3714 (3)	0.5262 (3)	0.0225 (12)	0.776 (4)
H5	0.6243	0.4014	0.4804	0.027*	0.776 (4)
N5'	0.6922 (10)	0.3397 (7)	0.5707 (7)	0.022*	0.224 (4)
H5'	0.7445	0.3164	0.6041	0.027*	0.224 (4)
N6	0.9823 (3)	0.4006 (2)	0.74805 (18)	0.0172 (8)	
H6	0.9958	0.3547	0.7498	0.021*	
N7	1.1956 (3)	0.2586 (2)	0.5087 (2)	0.0247 (9)	
H7	1.1641	0.2350	0.5500	0.030*	
N8	1.3126 (2)	0.16130 (15)	0.88166 (15)	0.0182 (8)	
H8	1.2556	0.1617	0.8610	0.022*	
C1	0.2940 (2)	0.70526 (15)	−0.00914 (15)	0.0241 (11)	
H1A	0.3335	0.7086	−0.0535	0.029*	
H1B	0.3119	0.6510	0.0300	0.029*	
C2	0.1789 (4)	0.7274 (3)	−0.0292 (3)	0.0345 (13)	
H2A	0.1617	0.6930	−0.0468	0.052*	
H2B	0.1609	0.7808	−0.0686	0.052*	
H2C	0.1393	0.7230	0.0148	0.052*	
C3	0.3195 (4)	0.8377 (3)	−0.0415 (2)	0.0246 (11)	
H3A	0.2479	0.8589	−0.0644	0.029*	
H3B	0.3319	0.8695	−0.0183	0.029*	
C4	0.3959 (4)	0.8441 (3)	−0.1014 (3)	0.0301 (12)	
H4A	0.3870	0.8982	−0.1387	0.045*	
H4B	0.3833	0.8135	−0.1252	0.045*	
H4C	0.4671	0.8246	−0.0794	0.045*	
C5	0.2721 (4)	0.7530 (3)	0.0870 (2)	0.0255 (11)	
H5A	0.2545	0.7019	0.1158	0.031*	
H5B	0.2062	0.7927	0.0720	0.031*	
C6	0.3388 (4)	0.7666 (3)	0.1360 (3)	0.0313 (12)	
H6A	0.3004	0.7646	0.1798	0.047*	
H6B	0.3556	0.8174	0.1079	0.047*	
H6C	0.4034	0.7267	0.1520	0.047*	
C7	0.0947 (3)	0.4578 (2)	0.0931 (2)	0.0198 (10)	
H7A	0.0323	0.4697	0.0616	0.024*	
H7B	0.0705	0.4556	0.1413	0.024*	
C8	0.1528 (3)	0.5219 (2)	0.0556 (2)	0.0207 (10)	
H8A	0.1067	0.5709	0.0482	0.031*	
H8B	0.1755	0.5253	0.0073	0.031*	
H8C	0.2138	0.5111	0.0871	0.031*	
C9	0.1908 (3)	0.3753 (3)	0.0361 (2)	0.0190 (10)	
H9A	0.2310	0.4151	0.0070	0.023*	
H9B	0.1270	0.3869	0.0060	0.023*	
C10	0.2556 (3)	0.2969 (2)	0.0498 (2)	0.0224 (10)	
H10A	0.2727	0.2968	0.0020	0.034*	
H10B	0.2160	0.2573	0.0783	0.034*	
H10C	0.3202	0.2860	0.0780	0.034*	
C11	0.1067 (3)	0.3176 (3)	0.1589 (2)	0.0201 (10)	
H11A	0.0975	0.3206	0.2065	0.024*	
H11B	0.1523	0.2668	0.1696	0.024*	
C12	0.0020 (3)	0.3225 (3)	0.1292 (3)	0.0255 (11)	
H12A	−0.0285	0.2805	0.1660	0.038*	
H12B	0.0105	0.3181	0.0828	0.038*	
H12C	−0.0443	0.3722	0.1196	0.038*	
C13	0.5780 (4)	0.0479 (3)	0.5879 (3)	0.0344 (13)	
H13A	0.5146	0.0466	0.5652	0.041*	
H13B	0.6384	0.0370	0.5599	0.041*	
C14	0.5679 (6)	0.1267 (3)	0.5816 (3)	0.065 (2)	
H14A	0.5576	0.1650	0.5290	0.098*	
H14B	0.6315	0.1288	0.6024	0.098*	
H14C	0.5079	0.1378	0.6090	0.098*	
C15	0.6393 (4)	−0.0902 (3)	0.6706 (3)	0.0300 (12)	
H15A	0.7041	−0.0872	0.6427	0.036*	
H15B	0.5901	−0.1041	0.6466	0.036*	
C16	0.6642 (4)	−0.1526 (3)	0.7503 (3)	0.0517 (17)	
H16A	0.6940	−0.2022	0.7506	0.078*	
H16B	0.6002	−0.1559	0.7780	0.078*	
H16C	0.7146	−0.1398	0.7738	0.078*	
C17	0.4948 (4)	−0.0111 (3)	0.7089 (3)	0.0249 (11)	
H17A	0.4680	0.0421	0.7023	0.030*	
H17B	0.5126	−0.0451	0.7625	0.030*	
C18	0.4092 (4)	−0.0359 (3)	0.6859 (3)	0.0282 (11)	
H18A	0.3484	−0.0327	0.7158	0.042*	
H18B	0.4339	−0.0891	0.6939	0.042*	
H18C	0.3897	−0.0019	0.6331	0.042*	
C19	0.8857 (6)	0.0872 (4)	0.4179 (5)	0.038 (3)	0.627 (10)
H19A	0.9280	0.0911	0.4541	0.046*	0.627 (10)
H19B	0.9276	0.0482	0.4039	0.046*	0.627 (10)
C19'	0.8708 (12)	0.0864 (6)	0.3795 (7)	0.038*	0.373 (10)
H19C	0.9462	0.0641	0.3808	0.046*	0.373 (10)
H19D	0.8315	0.0630	0.3587	0.046*	0.373 (10)
C20	0.7888 (7)	0.0626 (5)	0.4525 (5)	0.044 (3)	0.627 (10)
H20A	0.8066	0.0178	0.5015	0.066*	0.627 (10)
H20B	0.7542	0.0487	0.4207	0.066*	0.627 (10)
H20C	0.7419	0.1050	0.4583	0.066*	0.627 (10)
C20'	0.8358 (15)	0.0724 (9)	0.4570 (6)	0.044*	0.373 (10)
H20D	0.8511	0.0168	0.4904	0.066*	0.373 (10)
H20E	0.7607	0.0934	0.4548	0.066*	0.373 (10)
H20F	0.8732	0.0980	0.4756	0.066*	0.373 (10)
C21	0.8633 (9)	0.1551 (7)	0.2837 (5)	0.075 (4)	0.627 (10)
H21A	0.8868	0.2003	0.2444	0.090*	0.627 (10)
H21B	0.9183	0.1090	0.2917	0.090*	0.627 (10)
C21'	0.8587 (15)	0.1918 (11)	0.2574 (7)	0.075*	0.373 (10)
H21C	0.8605	0.2472	0.2306	0.090*	0.373 (10)
H21D	0.9263	0.1623	0.2508	0.090*	0.373 (10)
C22	0.7735 (9)	0.1488 (8)	0.2552 (7)	0.080 (4)	0.627 (10)
H22A	0.7938	0.1211	0.2255	0.120*	0.627 (10)
H22B	0.7318	0.2005	0.2239	0.120*	0.627 (10)
H22C	0.7321	0.1205	0.2965	0.120*	0.627 (10)
C22'	0.7774 (17)	0.1792 (15)	0.2239 (11)	0.080*	0.373 (10)
H22D	0.7818	0.2049	0.1696	0.120*	0.373 (10)
H22E	0.7095	0.2004	0.2376	0.120*	0.373 (10)
H22F	0.7851	0.1237	0.2414	0.120*	0.373 (10)
C23	0.9264 (8)	0.2132 (5)	0.3466 (6)	0.049 (3)	0.627 (10)
H23A	0.9102	0.2271	0.3878	0.059*	0.627 (10)
H23B	0.9083	0.2614	0.2994	0.059*	0.627 (10)
C23'	0.9174 (15)	0.2034 (10)	0.3591 (11)	0.049*	0.373 (10)
H23D	0.9125	0.1864	0.4132	0.059*	0.373 (10)
H23C	0.8959	0.2603	0.3329	0.059*	0.373 (10)
C24	1.0387 (8)	0.1828 (8)	0.3501 (8)	0.035 (2)	0.627 (10)
H24A	1.0751	0.2250	0.3343	0.053*	0.627 (10)
H24B	1.0547	0.1586	0.3171	0.053*	0.627 (10)
H24C	1.0616	0.1444	0.4013	0.053*	0.627 (10)
C24'	1.0264 (13)	0.1785 (15)	0.3423 (15)	0.035*	0.373 (10)
H24D	1.0718	0.2030	0.3562	0.053*	0.373 (10)
H24E	1.0305	0.1938	0.2889	0.053*	0.373 (10)
H24F	1.0488	0.1223	0.3705	0.053*	0.373 (10)
C25	0.6693 (4)	0.2917 (3)	0.5363 (3)	0.0262 (14)	0.776 (4)
H25A	0.6030	0.2818	0.5264	0.031*	0.776 (4)
H25B	0.6920	0.2556	0.5888	0.031*	0.776 (4)
C25'	0.7244 (14)	0.3481 (9)	0.4959 (7)	0.026*	0.224 (4)
H25C	0.7824	0.3755	0.4793	0.031*	0.224 (4)
H25D	0.6652	0.3777	0.4589	0.031*	0.224 (4)
C26	0.7509 (6)	0.2749 (4)	0.4863 (3)	0.0245 (17)	0.776 (4)
H26A	0.7596	0.2213	0.4958	0.037*	0.776 (4)
H26B	0.8174	0.2832	0.4966	0.037*	0.776 (4)
H26C	0.7283	0.3095	0.4341	0.037*	0.776 (4)
C26'	0.759 (3)	0.2677 (11)	0.5042 (13)	0.024*	0.224 (4)
H26D	0.7824	0.2700	0.4565	0.037*	0.224 (4)
H26E	0.7003	0.2419	0.5198	0.037*	0.224 (4)
H26F	0.8163	0.2389	0.5418	0.037*	0.224 (4)
C27	0.5682 (4)	0.3843 (3)	0.5754 (3)	0.0229 (14)	0.776 (4)
H27A	0.5521	0.4396	0.5634	0.028*	0.776 (4)
H27B	0.5041	0.3724	0.5647	0.028*	0.776 (4)
C27'	0.6069 (12)	0.2985 (9)	0.5915 (8)	0.023*	0.224 (4)
H27C	0.6325	0.2456	0.5973	0.028*	0.224 (4)
H27D	0.5503	0.3257	0.5522	0.028*	0.224 (4)
C28	0.5969 (5)	0.3376 (3)	0.6557 (3)	0.0261 (14)	0.776 (4)
H28A	0.5391	0.3490	0.6846	0.039*	0.776 (4)
H28B	0.6592	0.3500	0.6672	0.039*	0.776 (4)
H28C	0.6113	0.2827	0.6685	0.039*	0.776 (4)
C28'	0.5672 (14)	0.2959 (12)	0.6631 (9)	0.026*	0.224 (4)
H28D	0.5079	0.2707	0.6770	0.039*	0.224 (4)
H28E	0.5447	0.3485	0.6572	0.039*	0.224 (4)
H28F	0.6228	0.2666	0.7021	0.039*	0.224 (4)
C29	0.7450 (4)	0.3949 (3)	0.5310 (3)	0.0282 (15)	0.776 (4)
H29A	0.7930	0.3887	0.4935	0.034*	0.776 (4)
H29B	0.7796	0.3601	0.5807	0.034*	0.776 (4)
C29'	0.6463 (13)	0.4197 (7)	0.5591 (10)	0.028*	0.224 (4)
H29C	0.6188	0.4193	0.6060	0.034*	0.224 (4)
H29D	0.5888	0.4448	0.5206	0.034*	0.224 (4)
C30	0.7270 (6)	0.4775 (3)	0.5186 (4)	0.0205 (17)	0.776 (4)
H30A	0.7938	0.4890	0.5234	0.031*	0.776 (4)
H30B	0.6800	0.4842	0.5557	0.031*	0.776 (4)
H30C	0.6955	0.5126	0.4686	0.031*	0.776 (4)
C30'	0.734 (2)	0.4615 (11)	0.5342 (18)	0.020*	0.224 (4)
H30D	0.7079	0.5154	0.5240	0.031*	0.224 (4)
H30E	0.7620	0.4596	0.4889	0.031*	0.224 (4)
H30F	0.7893	0.4368	0.5735	0.031*	0.224 (4)
C31	1.1202 (4)	0.2836 (3)	0.4447 (3)	0.0368 (13)	
H31A	1.0891	0.2397	0.4533	0.044*	
H31B	1.1594	0.2962	0.3986	0.044*	
C32	1.0330 (4)	0.3525 (3)	0.4327 (3)	0.0363 (13)	
H32A	0.9873	0.3650	0.3904	0.054*	
H32B	1.0627	0.3969	0.4224	0.054*	
H32C	0.9925	0.3402	0.4776	0.054*	
C33	1.2265 (4)	0.3257 (3)	0.5115 (3)	0.0358 (13)	
H33A	1.2451	0.3607	0.4611	0.043*	
H33B	1.1655	0.3550	0.5256	0.043*	
C34	1.3170 (4)	0.3020 (3)	0.5656 (3)	0.0322 (12)	
H34A	1.3327	0.3481	0.5646	0.048*	
H34B	1.3783	0.2741	0.5514	0.048*	
H34C	1.2986	0.2685	0.6160	0.048*	
C35	1.2915 (4)	0.2011 (3)	0.5072 (3)	0.0377 (13)	
H35A	1.3293	0.1754	0.5563	0.045*	
H35B	1.3382	0.2295	0.4696	0.045*	
C36	1.2687 (4)	0.1394 (3)	0.4894 (3)	0.0302 (12)	
H36A	1.3344	0.1048	0.4892	0.045*	
H36B	1.2326	0.1641	0.4404	0.045*	
H36C	1.2244	0.1097	0.5273	0.045*	
C37	0.8857 (3)	0.4478 (3)	0.6989 (2)	0.0215 (10)	
H37A	0.9022	0.4600	0.6467	0.026*	
H37B	0.8639	0.4972	0.7016	0.026*	
C38	0.7960 (3)	0.4071 (3)	0.7203 (2)	0.0210 (10)	
H38A	0.7352	0.4404	0.6868	0.032*	
H38B	0.7783	0.3957	0.7716	0.032*	
H38C	0.8163	0.3589	0.7163	0.032*	
C39	1.0752 (3)	0.4353 (3)	0.7183 (2)	0.0218 (10)	
H39A	1.1335	0.4061	0.7561	0.026*	
H39B	1.0580	0.4889	0.7116	0.026*	
C40	1.1123 (4)	0.4367 (3)	0.6452 (2)	0.0275 (11)	
H40A	1.1731	0.4601	0.6304	0.041*	
H40B	1.0563	0.4669	0.6067	0.041*	
H40C	1.1316	0.3838	0.6513	0.041*	
C41	0.9646 (3)	0.3902 (3)	0.8260 (2)	0.0214 (10)	
H41A	0.9014	0.3691	0.8437	0.026*	
H41B	0.9510	0.4414	0.8250	0.026*	
C42	1.0544 (4)	0.3377 (3)	0.8808 (2)	0.0237 (10)	
H42A	1.0376	0.3344	0.9299	0.035*	
H42B	1.1173	0.3583	0.8641	0.035*	
H42C	1.0666	0.2861	0.8839	0.035*	
C43	1.3975 (3)	0.0997 (3)	0.8767 (2)	0.0229 (10)	
H43A	1.4086	0.1130	0.8237	0.027*	
H43B	1.4632	0.0982	0.8990	0.027*	
C44	1.3723 (4)	0.0214 (3)	0.9158 (3)	0.0293 (12)	
H44A	1.4298	−0.0167	0.9111	0.044*	
H44B	1.3081	0.0222	0.8933	0.044*	
H44C	1.3627	0.0074	0.9686	0.044*	
C45	1.3358 (4)	0.2403 (3)	0.8381 (2)	0.0264 (11)	
H45A	1.2883	0.2765	0.8531	0.032*	
H45B	1.4081	0.2380	0.8511	0.032*	
C46	1.3238 (5)	0.2706 (3)	0.7561 (3)	0.0462 (16)	
H46A	1.3387	0.3223	0.7310	0.069*	
H46B	1.2523	0.2734	0.7428	0.069*	
H46C	1.3727	0.2362	0.7406	0.069*	
C47	1.2892 (3)	0.1450 (3)	0.9610 (2)	0.0221 (10)	
H47A	1.2347	0.1882	0.9607	0.027*	
H47B	1.2612	0.0977	0.9853	0.027*	
C48	1.3837 (3)	0.1345 (3)	1.0059 (2)	0.0229 (10)	
H48A	1.3639	0.1244	1.0566	0.034*	
H48B	1.4112	0.1814	0.9827	0.034*	
H48C	1.4373	0.0908	1.0077	0.034*	

### Atomic displacement parameters (Å^2^)

**Table:**

	*U*^11^	*U*^22^	*U*^33^	*U*^12^	*U*^13^	*U*^23^
V1	0.0087 (4)	0.0141 (4)	0.0140 (3)	−0.0032 (3)	0.0005 (3)	−0.0071 (3)
V2	0.0081 (3)	0.0105 (4)	0.0087 (3)	−0.0013 (3)	0.0000 (3)	−0.0036 (3)
V3	0.0122 (4)	0.0115 (4)	0.0105 (3)	−0.0032 (3)	0.0004 (3)	−0.0025 (3)
V4	0.0090 (4)	0.0154 (4)	0.0115 (3)	−0.0037 (3)	0.0019 (3)	−0.0054 (3)
V5	0.0085 (3)	0.0097 (3)	0.0082 (3)	−0.0014 (3)	0.0002 (3)	−0.0036 (3)
V6	0.0117 (4)	0.0100 (4)	0.0119 (3)	−0.0028 (3)	−0.0003 (3)	−0.0048 (3)
V7	0.0083 (3)	0.0103 (3)	0.0087 (3)	−0.0025 (3)	0.0008 (3)	−0.0039 (3)
V8	0.0094 (3)	0.0104 (4)	0.0093 (3)	−0.0032 (3)	0.0013 (3)	−0.0045 (3)
V9	0.0117 (4)	0.0146 (4)	0.0100 (3)	−0.0037 (3)	0.0003 (3)	−0.0065 (3)
V10	0.0107 (4)	0.0140 (4)	0.0091 (3)	−0.0031 (3)	0.0020 (3)	−0.0050 (3)
V11	0.0086 (4)	0.0145 (4)	0.0154 (4)	−0.0034 (3)	0.0008 (3)	−0.0068 (3)
V12	0.0075 (3)	0.0091 (3)	0.0101 (3)	−0.0008 (3)	0.0004 (3)	−0.0037 (3)
V13	0.0120 (4)	0.0098 (4)	0.0112 (3)	−0.0026 (3)	0.0016 (3)	−0.0030 (3)
V14	0.0097 (4)	0.0122 (4)	0.0140 (4)	−0.0035 (3)	0.0018 (3)	−0.0053 (3)
V15	0.0075 (3)	0.0097 (4)	0.0120 (3)	−0.0023 (3)	0.0013 (3)	−0.0051 (3)
V16	0.0115 (4)	0.0103 (4)	0.0153 (4)	−0.0037 (3)	0.0014 (3)	−0.0056 (3)
V17	0.0111 (4)	0.0154 (4)	0.0147 (4)	−0.0050 (3)	0.0015 (3)	−0.0088 (3)
V18	0.0126 (4)	0.0142 (4)	0.0117 (3)	−0.0050 (3)	0.0029 (3)	−0.0064 (3)
V19	0.0088 (3)	0.0095 (3)	0.0097 (3)	−0.0017 (3)	0.0012 (3)	−0.0036 (3)
V20	0.0097 (3)	0.0090 (3)	0.0101 (3)	−0.0020 (3)	0.0002 (3)	−0.0040 (3)
O1	0.0113 (15)	0.0226 (17)	0.0195 (15)	−0.0052 (13)	0.0014 (12)	−0.0117 (14)
O2	0.0139 (15)	0.0157 (15)	0.0138 (14)	−0.0070 (12)	0.0037 (12)	−0.0081 (13)
O3	0.0166 (16)	0.0113 (15)	0.0135 (14)	−0.0017 (12)	0.0011 (12)	−0.0045 (12)
O4	0.0130 (15)	0.0115 (15)	0.0126 (14)	−0.0014 (12)	−0.0004 (11)	−0.0058 (12)
O5	0.0116 (15)	0.0160 (15)	0.0131 (14)	−0.0017 (12)	0.0002 (11)	−0.0085 (13)
O6	0.0126 (15)	0.0227 (17)	0.0168 (15)	−0.0061 (13)	0.0041 (12)	−0.0096 (14)
O7	0.0123 (15)	0.0164 (16)	0.0117 (14)	−0.0049 (12)	0.0004 (11)	−0.0046 (13)
O8	0.0208 (17)	0.0158 (16)	0.0158 (15)	−0.0063 (13)	0.0010 (12)	−0.0043 (13)
O9	0.0095 (14)	0.0117 (15)	0.0143 (14)	−0.0012 (12)	0.0000 (11)	−0.0058 (12)
O10	0.0111 (15)	0.0109 (14)	0.0132 (14)	−0.0003 (12)	−0.0001 (11)	−0.0054 (12)
O11	0.0096 (14)	0.0101 (14)	0.0097 (13)	−0.0036 (11)	0.0002 (11)	−0.0030 (12)
O12	0.0084 (14)	0.0105 (14)	0.0085 (13)	−0.0030 (11)	0.0007 (11)	−0.0031 (12)
O13	0.0108 (14)	0.0119 (15)	0.0094 (13)	−0.0020 (11)	0.0006 (11)	−0.0042 (12)
O14	0.0110 (15)	0.0143 (15)	0.0104 (14)	−0.0039 (12)	0.0029 (11)	−0.0059 (12)
O15	0.0137 (15)	0.0115 (15)	0.0072 (13)	−0.0033 (12)	0.0014 (11)	−0.0032 (12)
O16	0.0121 (15)	0.0137 (15)	0.0120 (14)	−0.0015 (12)	0.0019 (11)	−0.0068 (12)
O17	0.0074 (14)	0.0118 (14)	0.0073 (13)	−0.0031 (11)	−0.0011 (10)	−0.0024 (12)
O18	0.0135 (15)	0.0153 (15)	0.0142 (14)	−0.0036 (12)	0.0003 (12)	−0.0088 (13)
O19	0.0118 (15)	0.0096 (14)	0.0120 (14)	−0.0025 (11)	−0.0002 (11)	−0.0037 (12)
O20	0.0087 (14)	0.0118 (15)	0.0123 (14)	−0.0017 (11)	0.0007 (11)	−0.0065 (12)
O21	0.0119 (15)	0.0170 (16)	0.0148 (14)	−0.0037 (12)	−0.0005 (12)	−0.0082 (13)
O22	0.0097 (14)	0.0136 (15)	0.0153 (14)	−0.0022 (12)	0.0022 (11)	−0.0083 (13)
O23	0.0112 (15)	0.0135 (15)	0.0123 (14)	−0.0032 (12)	0.0013 (11)	−0.0079 (12)
O24	0.0152 (15)	0.0162 (16)	0.0122 (14)	−0.0048 (12)	0.0039 (12)	−0.0070 (13)
O25	0.0156 (16)	0.0133 (15)	0.0101 (14)	−0.0058 (12)	0.0027 (11)	−0.0020 (12)
O26	0.0162 (16)	0.0207 (16)	0.0103 (14)	−0.0051 (13)	0.0025 (12)	−0.0067 (13)
O27	0.0102 (14)	0.0145 (15)	0.0097 (13)	−0.0036 (12)	−0.0006 (11)	−0.0039 (12)
O28	0.0153 (16)	0.0216 (17)	0.0131 (14)	−0.0018 (13)	−0.0033 (12)	−0.0089 (13)
O29	0.0121 (15)	0.0151 (15)	0.0161 (15)	−0.0056 (12)	0.0041 (12)	−0.0081 (13)
O30	0.0118 (15)	0.0196 (16)	0.0212 (16)	−0.0058 (13)	0.0013 (12)	−0.0075 (14)
O31	0.0125 (15)	0.0147 (15)	0.0120 (14)	−0.0018 (12)	0.0005 (11)	−0.0066 (13)
O32	0.0167 (16)	0.0135 (15)	0.0166 (15)	−0.0032 (12)	0.0019 (12)	−0.0046 (13)
O33	0.0100 (14)	0.0118 (15)	0.0115 (14)	−0.0019 (12)	0.0012 (11)	−0.0034 (12)
O34	0.0112 (15)	0.0131 (15)	0.0131 (14)	−0.0037 (12)	0.0021 (11)	−0.0052 (12)
O35	0.0126 (15)	0.0163 (16)	0.0228 (16)	−0.0066 (12)	0.0036 (12)	−0.0099 (14)
O36	0.0121 (15)	0.0112 (15)	0.0176 (15)	−0.0024 (12)	0.0000 (12)	−0.0082 (13)
O37	0.0101 (14)	0.0100 (14)	0.0158 (14)	−0.0015 (11)	0.0003 (11)	−0.0057 (12)
O38	0.0156 (16)	0.0144 (16)	0.0254 (16)	−0.0056 (13)	0.0044 (13)	−0.0084 (14)
O39	0.0073 (14)	0.0111 (15)	0.0138 (14)	−0.0024 (11)	0.0015 (11)	−0.0058 (12)
O40	0.0069 (14)	0.0124 (15)	0.0142 (14)	−0.0027 (11)	0.0022 (11)	−0.0064 (12)
O41	0.0116 (15)	0.0160 (16)	0.0150 (14)	−0.0049 (12)	0.0011 (12)	−0.0072 (13)
O42	0.0222 (17)	0.0225 (17)	0.0185 (15)	−0.0071 (14)	0.0032 (13)	−0.0136 (14)
O43	0.0106 (15)	0.0154 (15)	0.0116 (14)	−0.0030 (12)	−0.0001 (11)	−0.0065 (12)
O44	0.0205 (17)	0.0201 (16)	0.0169 (15)	−0.0069 (13)	0.0042 (13)	−0.0117 (14)
O45	0.0134 (15)	0.0171 (16)	0.0094 (14)	−0.0049 (12)	0.0032 (11)	−0.0043 (12)
O46	0.0135 (15)	0.0156 (15)	0.0129 (14)	−0.0061 (12)	0.0043 (12)	−0.0063 (13)
O47	0.0123 (15)	0.0118 (15)	0.0130 (14)	−0.0045 (12)	0.0034 (11)	−0.0074 (12)
O48	0.0115 (15)	0.0148 (15)	0.0200 (15)	−0.0049 (12)	0.0023 (12)	−0.0104 (13)
O49	0.0109 (15)	0.0114 (15)	0.0133 (14)	−0.0002 (12)	0.0011 (11)	−0.0035 (12)
O50	0.0086 (14)	0.0088 (14)	0.0104 (13)	−0.0003 (11)	−0.0011 (11)	−0.0038 (12)
O51	0.0131 (15)	0.0105 (14)	0.0125 (14)	−0.0037 (12)	0.0029 (11)	−0.0050 (12)
O52	0.0096 (14)	0.0127 (15)	0.0148 (14)	−0.0048 (12)	0.0015 (11)	−0.0066 (12)
O53	0.0136 (15)	0.0140 (15)	0.0136 (14)	−0.0026 (12)	−0.0013 (11)	−0.0075 (13)
O54	0.0085 (14)	0.0094 (14)	0.0110 (13)	−0.0004 (11)	−0.0022 (11)	−0.0048 (12)
O55	0.0113 (15)	0.0135 (15)	0.0118 (14)	−0.0012 (12)	−0.0003 (11)	−0.0050 (12)
O56	0.0158 (15)	0.0087 (14)	0.0098 (14)	−0.0009 (12)	−0.0013 (11)	−0.0015 (12)
N1	0.0117 (18)	0.019 (2)	0.0218 (19)	−0.0027 (15)	0.0009 (15)	−0.0101 (17)
N2	0.0140 (19)	0.024 (2)	0.0158 (18)	−0.0059 (16)	0.0002 (15)	−0.0098 (17)
N3	0.019 (2)	0.017 (2)	0.0195 (19)	−0.0023 (16)	−0.0029 (15)	−0.0111 (17)
N4	0.031 (4)	0.048 (4)	0.030 (4)	0.010 (3)	−0.012 (3)	−0.012 (3)
N5	0.021 (2)	0.025 (3)	0.024 (2)	−0.005 (2)	0.003 (2)	−0.014 (2)
N6	0.022 (2)	0.0135 (19)	0.0182 (19)	−0.0040 (16)	0.0012 (15)	−0.0089 (16)
N7	0.030 (2)	0.028 (2)	0.0151 (19)	−0.0117 (19)	0.0109 (17)	−0.0086 (18)
N8	0.0159 (19)	0.0147 (19)	0.0193 (19)	−0.0046 (15)	−0.0026 (15)	−0.0029 (16)
C1	0.025 (3)	0.016 (2)	0.031 (3)	0.000 (2)	−0.010 (2)	−0.011 (2)
C2	0.029 (3)	0.025 (3)	0.040 (3)	−0.013 (2)	−0.013 (2)	−0.003 (2)
C3	0.027 (3)	0.018 (2)	0.029 (3)	−0.010 (2)	0.002 (2)	−0.009 (2)
C4	0.039 (3)	0.030 (3)	0.025 (3)	−0.013 (2)	0.005 (2)	−0.015 (2)
C5	0.024 (3)	0.022 (3)	0.025 (3)	0.000 (2)	0.007 (2)	−0.009 (2)
C6	0.032 (3)	0.037 (3)	0.027 (3)	0.005 (2)	0.007 (2)	−0.022 (2)
C7	0.017 (2)	0.022 (2)	0.018 (2)	0.0051 (19)	−0.0010 (18)	−0.012 (2)
C8	0.019 (2)	0.022 (2)	0.019 (2)	−0.0033 (19)	0.0032 (18)	−0.008 (2)
C9	0.018 (2)	0.025 (3)	0.014 (2)	−0.0072 (19)	0.0040 (18)	−0.009 (2)
C10	0.021 (3)	0.024 (3)	0.027 (3)	−0.009 (2)	0.008 (2)	−0.015 (2)
C11	0.017 (2)	0.028 (3)	0.016 (2)	−0.010 (2)	0.0028 (18)	−0.009 (2)
C12	0.017 (2)	0.028 (3)	0.036 (3)	−0.005 (2)	0.003 (2)	−0.018 (2)
C13	0.039 (3)	0.041 (3)	0.021 (3)	−0.014 (3)	−0.007 (2)	−0.009 (2)
C14	0.102 (6)	0.026 (3)	0.051 (4)	−0.025 (3)	−0.049 (4)	0.008 (3)
C15	0.017 (3)	0.029 (3)	0.054 (3)	−0.003 (2)	−0.001 (2)	−0.029 (3)
C16	0.040 (4)	0.024 (3)	0.086 (5)	0.002 (3)	−0.036 (3)	−0.020 (3)
C17	0.024 (3)	0.028 (3)	0.026 (3)	−0.002 (2)	0.001 (2)	−0.016 (2)
C18	0.020 (3)	0.036 (3)	0.037 (3)	−0.006 (2)	0.005 (2)	−0.023 (3)
C19	0.024 (4)	0.047 (4)	0.030 (4)	−0.012 (3)	0.007 (3)	−0.005 (3)
C20	0.030 (4)	0.049 (4)	0.036 (4)	−0.016 (3)	0.010 (3)	−0.004 (3)
C21	0.074 (6)	0.096 (6)	0.079 (5)	−0.012 (4)	0.012 (4)	−0.063 (5)
C22	0.073 (6)	0.077 (6)	0.110 (6)	−0.010 (4)	0.013 (4)	−0.063 (5)
C23	0.050 (5)	0.040 (4)	0.040 (5)	0.008 (3)	0.020 (4)	−0.013 (4)
C24	0.045 (4)	0.037 (4)	0.036 (4)	−0.017 (3)	−0.017 (3)	−0.022 (3)
C25	0.028 (3)	0.024 (3)	0.026 (3)	0.000 (2)	−0.006 (2)	−0.012 (2)
C26	0.029 (3)	0.032 (3)	0.015 (3)	0.001 (2)	−0.010 (3)	−0.015 (3)
C27	0.019 (3)	0.025 (3)	0.024 (3)	−0.002 (2)	0.002 (2)	−0.012 (2)
C28	0.026 (3)	0.028 (3)	0.025 (3)	−0.003 (2)	0.000 (2)	−0.014 (2)
C29	0.025 (3)	0.028 (3)	0.030 (3)	−0.001 (2)	0.002 (2)	−0.013 (3)
C30	0.021 (3)	0.015 (3)	0.020 (4)	0.001 (2)	−0.002 (2)	−0.006 (3)
C31	0.040 (3)	0.044 (3)	0.024 (3)	−0.015 (3)	0.005 (2)	−0.012 (3)
C32	0.045 (3)	0.028 (3)	0.025 (3)	−0.008 (3)	0.005 (2)	−0.002 (2)
C33	0.045 (4)	0.038 (3)	0.031 (3)	−0.021 (3)	0.019 (3)	−0.019 (3)
C34	0.035 (3)	0.038 (3)	0.032 (3)	−0.025 (3)	0.012 (2)	−0.017 (3)
C35	0.025 (3)	0.059 (4)	0.031 (3)	−0.009 (3)	0.007 (2)	−0.023 (3)
C36	0.032 (3)	0.036 (3)	0.028 (3)	−0.009 (2)	0.008 (2)	−0.019 (2)
C37	0.026 (3)	0.018 (2)	0.018 (2)	0.001 (2)	−0.0023 (19)	−0.009 (2)
C38	0.018 (2)	0.023 (3)	0.024 (2)	0.0036 (19)	−0.0025 (19)	−0.015 (2)
C39	0.021 (2)	0.025 (3)	0.022 (2)	−0.012 (2)	0.0060 (19)	−0.011 (2)
C40	0.024 (3)	0.035 (3)	0.028 (3)	−0.011 (2)	0.009 (2)	−0.017 (2)
C41	0.022 (2)	0.024 (3)	0.018 (2)	−0.003 (2)	0.0024 (19)	−0.010 (2)
C42	0.024 (3)	0.025 (3)	0.019 (2)	−0.008 (2)	0.0024 (19)	−0.007 (2)
C43	0.016 (2)	0.031 (3)	0.025 (2)	−0.002 (2)	0.0003 (19)	−0.016 (2)
C44	0.024 (3)	0.024 (3)	0.045 (3)	0.004 (2)	−0.008 (2)	−0.023 (3)
C45	0.031 (3)	0.021 (3)	0.027 (3)	−0.008 (2)	−0.004 (2)	−0.009 (2)
C46	0.075 (5)	0.031 (3)	0.034 (3)	−0.025 (3)	−0.014 (3)	−0.008 (3)
C47	0.020 (2)	0.023 (3)	0.024 (2)	−0.005 (2)	0.0017 (19)	−0.012 (2)
C48	0.023 (3)	0.028 (3)	0.017 (2)	−0.009 (2)	0.0019 (19)	−0.008 (2)

### Geometric parameters (Å, °)

**Table:**

V1—O1	1.596 (3)	C3—H3B	0.9900
V1—O2	1.805 (3)	C4—H4A	0.9800
V1—O21	1.850 (3)	C4—H4B	0.9800
V1—O13	1.945 (3)	C4—H4C	0.9800
V1—O10	2.028 (3)	C5—C6	1.516 (6)
V1—O11	2.338 (3)	C5—H5A	0.9900
V2—O10	1.688 (3)	C5—H5B	0.9900
V2—O9	1.691 (3)	C6—H6A	0.9800
V2—O27	1.890 (3)	C6—H6B	0.9800
V2—O20	1.948 (3)	C6—H6C	0.9800
V2—O12	2.091 (3)	C7—C8	1.510 (6)
V2—O11	2.148 (3)	C7—H7A	0.9900
V3—O8	1.602 (3)	C7—H7B	0.9900
V3—O25	1.807 (3)	C8—H8A	0.9800
V3—O7	1.834 (3)	C8—H8B	0.9800
V3—O19	1.953 (3)	C8—H8C	0.9800
V3—O9	2.055 (3)	C9—C10	1.514 (6)
V3—O12	2.395 (3)	C9—H9A	0.9900
V4—O6	1.611 (3)	C9—H9B	0.9900
V4—O7	1.813 (3)	C10—H10A	0.9800
V4—O14	1.846 (3)	C10—H10B	0.9800
V4—O24	1.897 (3)	C10—H10C	0.9800
V4—O5	2.076 (3)	C11—C12	1.506 (6)
V4—O12	2.328 (3)	C11—H11A	0.9900
V5—O5	1.680 (3)	C11—H11B	0.9900
V5—O4	1.692 (3)	C12—H12A	0.9800
V5—O23	1.842 (3)	C12—H12B	0.9800
V5—O17	2.055 (3)	C12—H12C	0.9800
V5—O12	2.070 (3)	C13—C14	1.496 (7)
V5—O11	2.124 (3)	C13—H13A	0.9900
V6—O3	1.615 (3)	C13—H13B	0.9900
V6—O22	1.773 (3)	C14—H14A	0.9800
V6—O2	1.849 (3)	C14—H14B	0.9800
V6—O15	1.997 (3)	C14—H14C	0.9800
V6—O4	2.021 (3)	C15—C16	1.518 (7)
V6—O11	2.230 (3)	C15—H15A	0.9900
V7—O16	1.617 (3)	C15—H15B	0.9900
V7—O13	1.728 (3)	C16—H16A	0.9800
V7—O20	1.900 (3)	C16—H16B	0.9800
V7—O15	1.945 (3)	C16—H16C	0.9800
V7—O17	2.122 (3)	C17—C18	1.504 (6)
V7—O11	2.200 (3)	C17—H17A	0.9900
V8—O18	1.622 (3)	C17—H17B	0.9900
V8—O19	1.733 (3)	C18—H18A	0.9800
V8—O14	1.859 (3)	C18—H18B	0.9800
V8—O20	1.991 (3)	C18—H18C	0.9800
V8—O17	2.123 (3)	C19—C20	1.488 (8)
V8—O12	2.223 (3)	C19—H19A	0.9900
V9—O28	1.605 (3)	C19—H19B	0.9900
V9—O21	1.797 (3)	C19'—C20'	1.519 (9)
V9—O22	1.875 (3)	C19'—H19C	0.9900
V9—O27	1.935 (3)	C19'—H19D	0.9900
V9—O23	2.024 (3)	C20—H20A	0.9800
V9—O11	2.338 (3)	C20—H20B	0.9800
V10—O26	1.607 (3)	C20—H20C	0.9800
V10—O24	1.776 (3)	C20'—H20D	0.9800
V10—O25	1.845 (3)	C20'—H20E	0.9800
V10—O23	1.996 (3)	C20'—H20F	0.9800
V10—O27	2.035 (3)	C21—C22	1.404 (8)
V10—O12	2.297 (3)	C21—H21A	0.9900
V11—O30	1.597 (3)	C21—H21B	0.9900
V11—O29	1.784 (3)	C21'—C22'	1.430 (10)
V11—O41	1.868 (3)	C21'—H21C	0.9900
V11—O49	1.938 (3)	C21'—H21D	0.9900
V11—O31	2.046 (3)	C22—H22A	0.9800
V11—O39	2.332 (3)	C22—H22B	0.9800
V12—O31	1.678 (3)	C22—H22C	0.9800
V12—O33	1.692 (3)	C22'—H22D	0.9800
V12—O43	1.900 (3)	C22'—H22E	0.9800
V12—O50	1.960 (3)	C22'—H22F	0.9800
V12—O40	2.106 (3)	C23—C24	1.460 (8)
V12—O39	2.112 (3)	C23—H23A	0.9900
V13—O32	1.600 (3)	C23—H23B	0.9900
V13—O45	1.813 (3)	C23'—C24'	1.481 (10)
V13—O34	1.832 (3)	C23'—H23D	0.9900
V13—O51	1.963 (3)	C23'—H23C	0.9900
V13—O33	2.018 (3)	C24—H24A	0.9800
V13—O40	2.367 (3)	C24—H24B	0.9800
V14—O35	1.595 (3)	C24—H24C	0.9800
V14—O34	1.822 (3)	C24'—H24D	0.9800
V14—O52	1.870 (3)	C24'—H24E	0.9800
V14—O46	1.880 (3)	C24'—H24F	0.9800
V14—O36	2.060 (3)	C25—C26	1.516 (7)
V14—O40	2.306 (3)	C25—H25A	0.9900
V15—O36	1.682 (3)	C25—H25B	0.9900
V15—O37	1.693 (3)	C25'—C26'	1.501 (10)
V15—O47	1.861 (3)	C25'—H25C	0.9900
V15—O54	2.050 (3)	C25'—H25D	0.9900
V15—O40	2.076 (3)	C26—H26A	0.9800
V15—O39	2.124 (3)	C26—H26B	0.9800
V16—O38	1.593 (3)	C26—H26C	0.9800
V16—O48	1.793 (3)	C26'—H26D	0.9800
V16—O29	1.853 (3)	C26'—H26E	0.9800
V16—O56	1.993 (3)	C26'—H26F	0.9800
V16—O37	2.015 (3)	C27—C28	1.478 (6)
V16—O39	2.284 (3)	C27—H27A	0.9900
V17—O42	1.594 (3)	C27—H27B	0.9900
V17—O41	1.815 (3)	C27'—C28'	1.490 (10)
V17—O48	1.868 (3)	C27'—H27C	0.9900
V17—O43	1.952 (3)	C27'—H27D	0.9900
V17—O47	2.006 (3)	C28—H28A	0.9800
V17—O39	2.328 (3)	C28—H28B	0.9800
V18—O44	1.606 (3)	C28—H28C	0.9800
V18—O46	1.796 (3)	C28'—H28D	0.9800
V18—O45	1.841 (3)	C28'—H28E	0.9800
V18—O47	1.999 (3)	C28'—H28F	0.9800
V18—O43	2.014 (3)	C29—C30	1.516 (6)
V18—O40	2.330 (3)	C29—H29A	0.9900
V19—O55	1.620 (3)	C29—H29B	0.9900
V19—O49	1.722 (3)	C29'—C30'	1.504 (10)
V19—O50	1.897 (3)	C29'—H29C	0.9900
V19—O56	1.940 (3)	C29'—H29D	0.9900
V19—O54	2.125 (3)	C30—H30A	0.9800
V19—O39	2.213 (3)	C30—H30B	0.9800
V20—O53	1.617 (3)	C30—H30C	0.9800
V20—O51	1.763 (3)	C30'—H30D	0.9800
V20—O52	1.830 (3)	C30'—H30E	0.9800
V20—O50	1.989 (3)	C30'—H30F	0.9800
V20—O54	2.114 (3)	C31—C32	1.516 (7)
V20—O40	2.214 (3)	C31—H31A	0.9900
O15—H15	0.8400	C31—H31B	0.9900
O17—H17	0.8400	C32—H32A	0.9800
O54—H54	0.8400	C32—H32B	0.9800
O56—H56	0.8400	C32—H32C	0.9800
N1—C5	1.506 (5)	C33—C34	1.512 (7)
N1—C1	1.508 (4)	C33—H33A	0.9900
N1—C3	1.509 (5)	C33—H33B	0.9900
N1—H1	0.8800	C34—H34A	0.9800
N2—C11	1.498 (5)	C34—H34B	0.9800
N2—C9	1.499 (5)	C34—H34C	0.9800
N2—C7	1.512 (5)	C35—C36	1.527 (7)
N2—H2	0.8800	C35—H35A	0.9900
N3—C13	1.487 (5)	C35—H35B	0.9900
N3—C15	1.497 (5)	C36—H36A	0.9800
N3—C17	1.499 (5)	C36—H36B	0.9800
N3—H3	0.8800	C36—H36C	0.9800
N4—C21	1.432 (8)	C37—C38	1.514 (6)
N4—C23	1.459 (7)	C37—H37A	0.9900
N4—C19	1.477 (7)	C37—H37B	0.9900
N4—H4	0.8800	C38—H38A	0.9800
N4'—C21'	1.439 (9)	C38—H38B	0.9800
N4'—C23'	1.455 (9)	C38—H38C	0.9800
N4'—C19'	1.461 (9)	C39—C40	1.519 (6)
N4'—H4'	0.8800	C39—H39A	0.9900
N5—C29	1.454 (6)	C39—H39B	0.9900
N5—C25	1.476 (5)	C40—H40A	0.9800
N5—C27	1.480 (5)	C40—H40B	0.9800
N5—H5	0.8800	C40—H40C	0.9800
N5'—C27'	1.469 (9)	C41—C42	1.508 (6)
N5'—C29'	1.489 (9)	C41—H41A	0.9900
N5'—C25'	1.489 (9)	C41—H41B	0.9900
N5'—H5'	0.8800	C42—H42A	0.9800
N6—C39	1.498 (5)	C42—H42B	0.9800
N6—C41	1.503 (5)	C42—H42C	0.9800
N6—C37	1.504 (5)	C43—C44	1.501 (6)
N6—H6	0.8800	C43—H43A	0.9900
N7—C31	1.504 (6)	C43—H43B	0.9900
N7—C35	1.513 (6)	C44—H44A	0.9800
N7—C33	1.515 (6)	C44—H44B	0.9800
N7—H7	0.8800	C44—H44C	0.9800
N8—C43	1.502 (5)	C45—C46	1.488 (6)
N8—C45	1.506 (5)	C45—H45A	0.9900
N8—C47	1.509 (5)	C45—H45B	0.9900
N8—H8	0.8800	C46—H46A	0.9800
C1—C2	1.508 (5)	C46—H46B	0.9800
C1—H1A	0.9900	C46—H46C	0.9800
C1—H1B	0.9900	C47—C48	1.508 (6)
C2—H2A	0.9800	C47—H47A	0.9900
C2—H2B	0.9800	C47—H47B	0.9900
C2—H2C	0.9800	C48—H48A	0.9800
C3—C4	1.503 (6)	C48—H48B	0.9800
C3—H3A	0.9900	C48—H48C	0.9800
			
O1—V1—O2	104.40 (14)	V16—O56—H56	123.4
O1—V1—O21	102.78 (13)	C5—N1—C1	112.7 (3)
O2—V1—O21	92.67 (12)	C5—N1—C3	111.9 (3)
O1—V1—O13	100.67 (13)	C1—N1—C3	113.1 (3)
O2—V1—O13	91.25 (11)	C5—N1—H1	106.2
O21—V1—O13	154.43 (12)	C1—N1—H1	106.2
O1—V1—O10	101.55 (13)	C3—N1—H1	106.2
O2—V1—O10	153.89 (12)	C11—N2—C9	114.1 (3)
O21—V1—O10	84.32 (11)	C11—N2—C7	109.8 (3)
O13—V1—O10	81.13 (11)	C9—N2—C7	112.6 (3)
O1—V1—O11	174.52 (12)	C11—N2—H2	106.6
O2—V1—O11	80.22 (11)	C9—N2—H2	106.6
O21—V1—O11	79.72 (11)	C7—N2—H2	106.6
O13—V1—O11	76.08 (10)	C13—N3—C15	110.5 (4)
O10—V1—O11	73.72 (10)	C13—N3—C17	112.6 (4)
O10—V2—O9	106.94 (13)	C15—N3—C17	113.9 (3)
O10—V2—O27	97.70 (12)	C13—N3—H3	106.4
O9—V2—O27	98.10 (12)	C15—N3—H3	106.4
O10—V2—O20	96.18 (12)	C17—N3—H3	106.4
O9—V2—O20	96.89 (12)	C21—N4—C23	109.9 (6)
O27—V2—O20	155.62 (12)	C21—N4—C19	112.3 (7)
O10—V2—O12	163.29 (12)	C23—N4—C19	111.6 (5)
O9—V2—O12	89.61 (12)	C21—N4—H4	107.6
O27—V2—O12	81.83 (11)	C23—N4—H4	107.6
O20—V2—O12	79.16 (10)	C19—N4—H4	107.6
O10—V2—O11	85.72 (12)	C21'—N4'—C23'	110.0 (8)
O9—V2—O11	167.15 (12)	C21'—N4'—C19'	110.8 (9)
O27—V2—O11	81.93 (11)	C23'—N4'—C19'	110.7 (8)
O20—V2—O11	79.22 (10)	C21'—N4'—H4'	108.4
O12—V2—O11	77.66 (10)	C23'—N4'—H4'	108.4
O8—V3—O25	103.01 (13)	C19'—N4'—H4'	108.4
O8—V3—O7	104.72 (14)	C29—N5—C25	113.2 (4)
O25—V3—O7	93.32 (12)	C29—N5—C27	113.9 (4)
O8—V3—O19	102.39 (13)	C25—N5—C27	110.9 (4)
O25—V3—O19	153.24 (12)	C29—N5—H5	106.0
O7—V3—O19	88.24 (11)	C25—N5—H5	106.0
O8—V3—O9	100.74 (13)	C27—N5—H5	106.0
O25—V3—O9	85.47 (11)	C27'—N5'—C29'	106.8 (8)
O7—V3—O9	154.09 (12)	C27'—N5'—C25'	107.2 (8)
O19—V3—O9	81.69 (11)	C29'—N5'—C25'	105.1 (8)
O8—V3—O12	173.91 (13)	C27'—N5'—H5'	112.4
O25—V3—O12	79.06 (10)	C29'—N5'—H5'	112.4
O7—V3—O12	80.75 (11)	C25'—N5'—H5'	112.4
O19—V3—O12	74.84 (10)	C39—N6—C41	110.2 (3)
O9—V3—O12	73.61 (10)	C39—N6—C37	112.1 (3)
O6—V4—O7	104.97 (14)	C41—N6—C37	110.7 (3)
O6—V4—O14	101.65 (13)	C39—N6—H6	107.9
O7—V4—O14	92.98 (12)	C41—N6—H6	107.9
O6—V4—O24	101.85 (13)	C37—N6—H6	107.9
O7—V4—O24	90.39 (12)	C31—N7—C35	111.1 (4)
O14—V4—O24	154.50 (12)	C31—N7—C33	112.9 (4)
O6—V4—O5	99.00 (13)	C35—N7—C33	110.4 (4)
O7—V4—O5	155.89 (12)	C31—N7—H7	107.4
O14—V4—O5	84.62 (11)	C35—N7—H7	107.4
O24—V4—O5	82.08 (11)	C33—N7—H7	107.4
O6—V4—O12	171.96 (13)	C43—N8—C45	111.9 (3)
O7—V4—O12	83.06 (11)	C43—N8—C47	112.7 (3)
O14—V4—O12	77.85 (10)	C45—N8—C47	110.9 (3)
O24—V4—O12	77.49 (10)	C43—N8—H8	107.0
O5—V4—O12	72.97 (10)	C45—N8—H8	107.0
O5—V5—O4	106.57 (13)	C47—N8—H8	107.0
O5—V5—O23	101.53 (12)	C2—C1—N1	112.8 (3)
O4—V5—O23	99.54 (12)	C2—C1—H1A	109.0
O5—V5—O17	93.84 (11)	N1—C1—H1A	109.0
O4—V5—O17	93.43 (11)	C2—C1—H1B	109.0
O23—V5—O17	156.06 (12)	N1—C1—H1B	109.0
O5—V5—O12	88.25 (12)	H1A—C1—H1B	107.8
O4—V5—O12	163.32 (12)	C1—C2—H2A	109.5
O23—V5—O12	84.53 (11)	C1—C2—H2B	109.5
O17—V5—O12	77.65 (10)	H2A—C2—H2B	109.5
O5—V5—O11	165.37 (12)	C1—C2—H2C	109.5
O4—V5—O11	85.68 (12)	H2A—C2—H2C	109.5
O23—V5—O11	83.82 (11)	H2B—C2—H2C	109.5
O17—V5—O11	77.15 (10)	C4—C3—N1	113.2 (4)
O12—V5—O11	78.64 (10)	C4—C3—H3A	108.9
O3—V6—O22	105.10 (13)	N1—C3—H3A	108.9
O3—V6—O2	105.55 (13)	C4—C3—H3B	108.9
O22—V6—O2	93.48 (12)	N1—C3—H3B	108.9
O3—V6—O15	96.42 (12)	H3A—C3—H3B	107.8
O22—V6—O15	157.47 (12)	C3—C4—H4A	109.5
O2—V6—O15	86.99 (11)	C3—C4—H4B	109.5
O3—V6—O4	95.51 (13)	H4A—C4—H4B	109.5
O22—V6—O4	88.42 (11)	C3—C4—H4C	109.5
O2—V6—O4	157.54 (12)	H4A—C4—H4C	109.5
O15—V6—O4	82.89 (11)	H4B—C4—H4C	109.5
O3—V6—O11	168.28 (12)	N1—C5—C6	111.9 (4)
O22—V6—O11	82.70 (11)	N1—C5—H5A	109.2
O2—V6—O11	82.32 (11)	C6—C5—H5A	109.2
O15—V6—O11	75.03 (10)	N1—C5—H5B	109.2
O4—V6—O11	75.72 (10)	C6—C5—H5B	109.2
O16—V7—O13	105.63 (13)	H5A—C5—H5B	107.9
O16—V7—O20	103.96 (12)	C5—C6—H6A	109.5
O13—V7—O20	97.78 (12)	C5—C6—H6B	109.5
O16—V7—O15	97.43 (12)	H6A—C6—H6B	109.5
O13—V7—O15	94.28 (12)	C5—C6—H6C	109.5
O20—V7—O15	151.50 (11)	H6A—C6—H6C	109.5
O16—V7—O17	96.17 (12)	H6B—C6—H6C	109.5
O13—V7—O17	158.17 (11)	C8—C7—N2	113.1 (3)
O20—V7—O17	75.42 (11)	C8—C7—H7A	109.0
O15—V7—O17	83.84 (11)	N2—C7—H7A	109.0
O16—V7—O11	169.05 (12)	C8—C7—H7B	109.0
O13—V7—O11	84.23 (11)	N2—C7—H7B	109.0
O20—V7—O11	78.92 (10)	H7A—C7—H7B	107.8
O15—V7—O11	76.73 (10)	C7—C8—H8A	109.5
O17—V7—O11	74.15 (10)	C7—C8—H8B	109.5
O18—V8—O19	107.20 (13)	H8A—C8—H8B	109.5
O18—V8—O14	101.55 (13)	C7—C8—H8C	109.5
O19—V8—O14	97.62 (12)	H8A—C8—H8C	109.5
O18—V8—O20	99.80 (12)	H8B—C8—H8C	109.5
O19—V8—O20	93.20 (12)	N2—C9—C10	112.7 (3)
O14—V8—O20	151.95 (11)	N2—C9—H9A	109.1
O18—V8—O17	95.59 (12)	C10—C9—H9A	109.1
O19—V8—O17	155.43 (11)	N2—C9—H9B	109.1
O14—V8—O17	86.38 (11)	C10—C9—H9B	109.1
O20—V8—O17	73.58 (10)	H9A—C9—H9B	107.8
O18—V8—O12	168.37 (12)	C9—C10—H10A	109.5
O19—V8—O12	83.73 (11)	C9—C10—H10B	109.5
O14—V8—O12	80.41 (10)	H10A—C10—H10B	109.5
O20—V8—O12	75.13 (10)	C9—C10—H10C	109.5
O17—V8—O12	73.00 (10)	H10A—C10—H10C	109.5
O28—V9—O21	103.48 (13)	H10B—C10—H10C	109.5
O28—V9—O22	103.92 (13)	N2—C11—C12	113.8 (4)
O21—V9—O22	93.07 (12)	N2—C11—H11A	108.8
O28—V9—O27	101.40 (13)	C12—C11—H11A	108.8
O21—V9—O27	92.61 (12)	N2—C11—H11B	108.8
O22—V9—O27	151.94 (11)	C12—C11—H11B	108.8
O28—V9—O23	100.91 (13)	H11A—C11—H11B	107.7
O21—V9—O23	154.82 (12)	C11—C12—H12A	109.5
O22—V9—O23	87.04 (11)	C11—C12—H12B	109.5
O27—V9—O23	76.43 (11)	H12A—C12—H12B	109.5
O28—V9—O11	175.30 (13)	C11—C12—H12C	109.5
O21—V9—O11	80.74 (11)	H12A—C12—H12C	109.5
O22—V9—O11	77.70 (10)	H12B—C12—H12C	109.5
O27—V9—O11	76.15 (10)	N3—C13—C14	112.1 (4)
O23—V9—O11	74.68 (10)	N3—C13—H13A	109.2
O26—V10—O24	105.10 (13)	C14—C13—H13A	109.2
O26—V10—O25	103.03 (13)	N3—C13—H13B	109.2
O24—V10—O25	94.98 (13)	C14—C13—H13B	109.2
O26—V10—O23	99.23 (13)	H13A—C13—H13B	107.9
O24—V10—O23	92.22 (12)	C13—C14—H14A	109.5
O25—V10—O23	153.89 (12)	C13—C14—H14B	109.5
O26—V10—O27	99.72 (13)	H14A—C14—H14B	109.5
O24—V10—O27	153.59 (11)	C13—C14—H14C	109.5
O25—V10—O27	88.01 (12)	H14A—C14—H14C	109.5
O23—V10—O27	74.84 (11)	H14B—C14—H14C	109.5
O26—V10—O12	172.44 (12)	N3—C15—C16	111.9 (4)
O24—V10—O12	80.67 (11)	N3—C15—H15A	109.2
O25—V10—O12	81.03 (11)	C16—C15—H15A	109.2
O23—V10—O12	75.42 (10)	N3—C15—H15B	109.2
O27—V10—O12	73.87 (10)	C16—C15—H15B	109.2
O30—V11—O29	104.27 (14)	H15A—C15—H15B	107.9
O30—V11—O41	101.57 (13)	C15—C16—H16A	109.5
O29—V11—O41	92.07 (12)	C15—C16—H16B	109.5
O30—V11—O49	101.62 (13)	H16A—C16—H16B	109.5
O29—V11—O49	91.83 (12)	C15—C16—H16C	109.5
O41—V11—O49	154.74 (12)	H16A—C16—H16C	109.5
O30—V11—O31	101.81 (13)	H16B—C16—H16C	109.5
O29—V11—O31	153.90 (12)	N3—C17—C18	114.6 (4)
O41—V11—O31	83.68 (11)	N3—C17—H17A	108.6
O49—V11—O31	81.89 (11)	C18—C17—H17A	108.6
O30—V11—O39	175.03 (12)	N3—C17—H17B	108.6
O29—V11—O39	80.54 (11)	C18—C17—H17B	108.6
O41—V11—O39	79.32 (10)	H17A—C17—H17B	107.6
O49—V11—O39	76.73 (10)	C17—C18—H18A	109.5
O31—V11—O39	73.37 (10)	C17—C18—H18B	109.5
O31—V12—O33	107.24 (13)	H18A—C18—H18B	109.5
O31—V12—O43	97.90 (12)	C17—C18—H18C	109.5
O33—V12—O43	98.20 (12)	H18A—C18—H18C	109.5
O31—V12—O50	96.89 (12)	H18B—C18—H18C	109.5
O33—V12—O50	95.29 (12)	N4—C19—C20	110.1 (6)
O43—V12—O50	156.03 (12)	N4—C19—H19A	109.6
O31—V12—O40	164.78 (12)	C20—C19—H19A	109.6
O33—V12—O40	87.76 (12)	N4—C19—H19B	109.6
O43—V12—O40	82.09 (11)	C20—C19—H19B	109.6
O50—V12—O40	78.72 (10)	H19A—C19—H19B	108.2
O31—V12—O39	86.90 (12)	N4'—C19'—C20'	104.9 (8)
O33—V12—O39	165.51 (12)	N4'—C19'—H19C	110.8
O43—V12—O39	82.54 (11)	C20'—C19'—H19C	110.8
O50—V12—O39	79.54 (10)	N4'—C19'—H19D	110.8
O40—V12—O39	77.98 (10)	C20'—C19'—H19D	110.8
O32—V13—O45	102.99 (13)	H19C—C19'—H19D	108.8
O32—V13—O34	104.03 (14)	C19—C20—H20A	109.5
O45—V13—O34	92.07 (12)	C19—C20—H20B	109.5
O32—V13—O51	100.42 (13)	H20A—C20—H20B	109.5
O45—V13—O51	155.67 (12)	C19—C20—H20C	109.5
O34—V13—O51	88.62 (12)	H20A—C20—H20C	109.5
O32—V13—O33	101.13 (13)	H20B—C20—H20C	109.5
O45—V13—O33	86.70 (12)	C19'—C20'—H20D	109.5
O34—V13—O33	154.43 (12)	C19'—C20'—H20E	109.5
O51—V13—O33	82.36 (11)	H20D—C20'—H20E	109.5
O32—V13—O40	174.28 (12)	C19'—C20'—H20F	109.5
O45—V13—O40	79.72 (11)	H20D—C20'—H20F	109.5
O34—V13—O40	80.77 (11)	H20E—C20'—H20F	109.5
O51—V13—O40	76.38 (10)	C22—C21—N4	119.9 (8)
O33—V13—O40	73.87 (10)	C22—C21—H21A	107.4
O35—V14—O34	103.00 (13)	N4—C21—H21A	107.4
O35—V14—O52	102.32 (13)	C22—C21—H21B	107.4
O34—V14—O52	92.20 (12)	N4—C21—H21B	107.4
O35—V14—O46	101.73 (13)	H21A—C21—H21B	106.9
O34—V14—O46	89.85 (12)	C22'—C21'—N4'	114.6 (10)
O52—V14—O46	154.73 (12)	C22'—C21'—H21C	108.6
O35—V14—O36	100.53 (13)	N4'—C21'—H21C	108.6
O34—V14—O36	156.38 (12)	C22'—C21'—H21D	108.6
O52—V14—O36	84.77 (11)	N4'—C21'—H21D	108.6
O46—V14—O36	83.35 (11)	H21C—C21'—H21D	107.6
O35—V14—O40	174.30 (13)	C21—C22—H22A	109.5
O34—V14—O40	82.70 (11)	C21—C22—H22B	109.5
O52—V14—O40	76.94 (10)	H22A—C22—H22B	109.5
O46—V14—O40	78.35 (11)	C21—C22—H22C	109.5
O36—V14—O40	73.79 (10)	H22A—C22—H22C	109.5
O36—V15—O37	106.43 (13)	H22B—C22—H22C	109.5
O36—V15—O47	100.70 (12)	C21'—C22'—H22D	109.5
O37—V15—O47	99.52 (12)	C21'—C22'—H22E	109.5
O36—V15—O54	94.43 (12)	H22D—C22'—H22E	109.5
O37—V15—O54	93.40 (12)	C21'—C22'—H22F	109.5
O47—V15—O54	156.37 (12)	H22D—C22'—H22F	109.5
O36—V15—O40	88.13 (12)	H22E—C22'—H22F	109.5
O37—V15—O40	163.68 (12)	N4—C23—C24	116.7 (7)
O47—V15—O40	84.58 (11)	N4—C23—H23A	108.1
O54—V15—O40	77.84 (10)	C24—C23—H23A	108.1
O36—V15—O39	165.37 (12)	N4—C23—H23B	108.1
O37—V15—O39	86.34 (12)	C24—C23—H23B	108.1
O47—V15—O39	83.75 (11)	H23A—C23—H23B	107.3
O54—V15—O39	77.38 (10)	N4'—C23'—C24'	110.0 (9)
O40—V15—O39	78.36 (10)	N4'—C23'—H23D	109.7
O38—V16—O48	105.15 (14)	C24'—C23'—H23D	109.7
O38—V16—O29	103.52 (14)	N4'—C23'—H23C	109.7
O48—V16—O29	92.80 (12)	C24'—C23'—H23C	109.7
O38—V16—O56	98.58 (13)	H23D—C23'—H23C	108.2
O48—V16—O56	155.65 (12)	C23—C24—H24A	109.5
O29—V16—O56	86.83 (11)	C23—C24—H24B	109.5
O38—V16—O37	100.13 (13)	H24A—C24—H24B	109.5
O48—V16—O37	87.75 (12)	C23—C24—H24C	109.5
O29—V16—O37	155.31 (12)	H24A—C24—H24C	109.5
O56—V16—O37	82.72 (11)	H24B—C24—H24C	109.5
O38—V16—O39	171.78 (13)	C23'—C24'—H24D	109.5
O48—V16—O39	81.61 (11)	C23'—C24'—H24E	109.5
O29—V16—O39	80.49 (11)	H24D—C24'—H24E	109.5
O56—V16—O39	74.31 (10)	C23'—C24'—H24F	109.5
O37—V16—O39	75.17 (10)	H24D—C24'—H24F	109.5
O42—V17—O41	102.84 (14)	H24E—C24'—H24F	109.5
O42—V17—O48	102.41 (14)	N5—C25—C26	113.8 (5)
O41—V17—O48	93.44 (12)	N5—C25—H25A	108.8
O42—V17—O43	102.11 (13)	C26—C25—H25A	108.8
O41—V17—O43	91.65 (12)	N5—C25—H25B	108.8
O48—V17—O43	153.15 (12)	C26—C25—H25B	108.8
O42—V17—O47	100.97 (13)	H25A—C25—H25B	107.7
O41—V17—O47	155.28 (12)	N5'—C25'—C26'	106.1 (9)
O48—V17—O47	87.87 (11)	N5'—C25'—H25C	110.5
O43—V17—O47	76.87 (11)	C26'—C25'—H25C	110.5
O42—V17—O39	176.32 (13)	N5'—C25'—H25D	110.5
O41—V17—O39	80.45 (11)	C26'—C25'—H25D	110.5
O48—V17—O39	78.90 (10)	H25C—C25'—H25D	108.7
O43—V17—O39	75.99 (10)	C25—C26—H26A	109.5
O47—V17—O39	75.58 (10)	C25—C26—H26B	109.5
O44—V18—O46	104.23 (14)	H26A—C26—H26B	109.5
O44—V18—O45	102.53 (13)	C25—C26—H26C	109.5
O46—V18—O45	93.58 (12)	H26A—C26—H26C	109.5
O44—V18—O47	101.47 (13)	H26B—C26—H26C	109.5
O46—V18—O47	91.17 (12)	C25'—C26'—H26D	109.5
O45—V18—O47	153.55 (11)	C25'—C26'—H26E	109.5
O44—V18—O43	101.83 (13)	H26D—C26'—H26E	109.5
O46—V18—O43	152.67 (12)	C25'—C26'—H26F	109.5
O45—V18—O43	88.69 (12)	H26D—C26'—H26F	109.5
O47—V18—O43	75.62 (11)	H26E—C26'—H26F	109.5
O44—V18—O40	175.31 (12)	C28—C27—N5	114.1 (4)
O46—V18—O40	79.29 (11)	C28—C27—H27A	108.7
O45—V18—O40	80.19 (10)	N5—C27—H27A	108.7
O47—V18—O40	75.17 (10)	C28—C27—H27B	108.7
O43—V18—O40	74.28 (10)	N5—C27—H27B	108.7
O55—V19—O49	104.51 (13)	H27A—C27—H27B	107.6
O55—V19—O50	102.61 (12)	N5'—C27'—C28'	107.9 (9)
O49—V19—O50	98.40 (12)	N5'—C27'—H27C	110.1
O55—V19—O56	99.35 (12)	C28'—C27'—H27C	110.1
O49—V19—O56	94.41 (12)	N5'—C27'—H27D	110.1
O50—V19—O56	150.82 (12)	C28'—C27'—H27D	110.1
O55—V19—O54	97.27 (12)	H27C—C27'—H27D	108.4
O49—V19—O54	158.17 (11)	C27—C28—H28A	109.5
O50—V19—O54	75.18 (11)	C27—C28—H28B	109.5
O56—V19—O54	83.22 (11)	H28A—C28—H28B	109.5
O55—V19—O39	170.72 (12)	C27—C28—H28C	109.5
O49—V19—O39	84.37 (11)	H28A—C28—H28C	109.5
O50—V19—O39	78.33 (10)	H28B—C28—H28C	109.5
O56—V19—O39	76.98 (10)	C27'—C28'—H28D	109.5
O54—V19—O39	73.94 (10)	C27'—C28'—H28E	109.5
O53—V20—O51	106.05 (13)	H28D—C28'—H28E	109.5
O53—V20—O52	101.71 (13)	C27'—C28'—H28F	109.5
O51—V20—O52	96.88 (12)	H28D—C28'—H28F	109.5
O53—V20—O50	99.68 (12)	H28E—C28'—H28F	109.5
O51—V20—O50	93.84 (12)	N5—C29—C30	113.9 (5)
O52—V20—O50	152.33 (11)	N5—C29—H29A	108.8
O53—V20—O54	95.49 (12)	C30—C29—H29A	108.8
O51—V20—O54	156.75 (11)	N5—C29—H29B	108.8
O52—V20—O54	87.01 (11)	C30—C29—H29B	108.8
O50—V20—O54	73.62 (10)	H29A—C29—H29B	107.7
O53—V20—O40	168.82 (12)	N5'—C29'—C30'	105.6 (9)
O51—V20—O40	84.53 (11)	N5'—C29'—H29C	110.6
O52—V20—O40	80.16 (11)	C30'—C29'—H29C	110.6
O50—V20—O40	75.55 (10)	N5'—C29'—H29D	110.6
O54—V20—O40	73.53 (10)	C30'—C29'—H29D	110.6
V1—O2—V6	113.17 (14)	H29C—C29'—H29D	108.8
V5—O4—V6	109.31 (14)	C29—C30—H30A	109.5
V5—O5—V4	109.84 (14)	C29—C30—H30B	109.5
V4—O7—V3	114.90 (14)	H30A—C30—H30B	109.5
V2—O9—V3	110.17 (14)	C29—C30—H30C	109.5
V2—O10—V1	112.93 (14)	H30A—C30—H30C	109.5
V5—O11—V2	99.73 (11)	H30B—C30—H30C	109.5
V5—O11—V7	101.37 (11)	C29'—C30'—H30D	109.5
V2—O11—V7	90.47 (10)	C29'—C30'—H30E	109.5
V5—O11—V6	88.33 (10)	H30D—C30'—H30E	109.5
V2—O11—V6	169.28 (13)	C29'—C30'—H30F	109.5
V7—O11—V6	94.88 (10)	H30D—C30'—H30F	109.5
V5—O11—V9	90.21 (9)	H30E—C30'—H30F	109.5
V2—O11—V9	88.57 (9)	N7—C31—C32	114.4 (4)
V7—O11—V9	168.37 (13)	N7—C31—H31A	108.7
V6—O11—V9	84.31 (9)	C32—C31—H31A	108.7
V5—O11—V1	169.96 (13)	N7—C31—H31B	108.7
V2—O11—V1	87.43 (9)	C32—C31—H31B	108.7
V7—O11—V1	85.49 (9)	H31A—C31—H31B	107.6
V6—O11—V1	83.75 (9)	C31—C32—H32A	109.5
V9—O11—V1	82.88 (8)	C31—C32—H32B	109.5
V5—O12—V2	103.41 (11)	H32A—C32—H32B	109.5
V5—O12—V8	101.39 (10)	C31—C32—H32C	109.5
V2—O12—V8	94.15 (10)	H32A—C32—H32C	109.5
V5—O12—V10	89.55 (10)	H32B—C32—H32C	109.5
V2—O12—V10	92.89 (10)	C34—C33—N7	113.8 (4)
V8—O12—V10	165.24 (13)	C34—C33—H33A	108.8
V5—O12—V4	88.76 (10)	N7—C33—H33A	108.8
V2—O12—V4	167.59 (13)	C34—C33—H33B	108.8
V8—O12—V4	85.69 (9)	N7—C33—H33B	108.8
V10—O12—V4	84.66 (9)	H33A—C33—H33B	107.7
V5—O12—V3	167.75 (13)	C33—C34—H34A	109.5
V2—O12—V3	86.41 (9)	C33—C34—H34B	109.5
V8—O12—V3	84.94 (9)	H34A—C34—H34B	109.5
V10—O12—V3	82.57 (8)	C33—C34—H34C	109.5
V4—O12—V3	81.21 (8)	H34A—C34—H34C	109.5
V7—O13—V1	113.93 (14)	H34B—C34—H34C	109.5
V4—O14—V8	113.36 (14)	N7—C35—C36	114.4 (4)
V7—O15—V6	111.75 (12)	N7—C35—H35A	108.7
V7—O15—H15	124.1	C36—C35—H35A	108.7
V6—O15—H15	124.1	N7—C35—H35B	108.7
V5—O17—V7	106.45 (11)	C36—C35—H35B	108.7
V5—O17—V8	105.39 (11)	H35A—C35—H35B	107.6
V7—O17—V8	96.11 (11)	C35—C36—H36A	109.5
V5—O17—H17	115.6	C35—C36—H36B	109.5
V7—O17—H17	115.6	H36A—C36—H36B	109.5
V8—O17—H17	115.6	C35—C36—H36C	109.5
V8—O19—V3	115.53 (14)	H36A—C36—H36C	109.5
V7—O20—V2	106.70 (12)	H36B—C36—H36C	109.5
V7—O20—V8	108.45 (13)	N6—C37—C38	112.9 (4)
V2—O20—V8	106.69 (12)	N6—C37—H37A	109.0
V9—O21—V1	116.12 (14)	C38—C37—H37A	109.0
V6—O22—V9	114.37 (14)	N6—C37—H37B	109.0
V5—O23—V10	106.66 (13)	C38—C37—H37B	109.0
V5—O23—V9	109.81 (13)	H37A—C37—H37B	107.8
V10—O23—V9	99.20 (12)	C37—C38—H38A	109.5
V10—O24—V4	115.97 (14)	C37—C38—H38B	109.5
V3—O25—V10	115.91 (14)	H38A—C38—H38B	109.5
V2—O27—V9	110.10 (13)	C37—C38—H38C	109.5
V2—O27—V10	108.32 (12)	H38A—C38—H38C	109.5
V9—O27—V10	100.89 (12)	H38B—C38—H38C	109.5
V11—O29—V16	115.20 (14)	N6—C39—C40	115.4 (4)
V12—O31—V11	111.69 (14)	N6—C39—H39A	108.4
V12—O33—V13	111.47 (14)	C40—C39—H39A	108.4
V14—O34—V13	114.32 (14)	N6—C39—H39B	108.4
V15—O36—V14	109.37 (14)	C40—C39—H39B	108.4
V15—O37—V16	110.25 (14)	H39A—C39—H39B	107.5
V12—O39—V15	100.64 (11)	C39—C40—H40A	109.5
V12—O39—V19	90.95 (10)	C39—C40—H40B	109.5
V15—O39—V19	101.05 (11)	H40A—C40—H40B	109.5
V12—O39—V16	169.70 (14)	C39—C40—H40C	109.5
V15—O39—V16	87.41 (9)	H40A—C40—H40C	109.5
V19—O39—V16	93.81 (10)	H40B—C40—H40C	109.5
V12—O39—V17	89.64 (10)	N6—C41—C42	114.2 (4)
V15—O39—V17	89.89 (9)	N6—C41—H41A	108.7
V19—O39—V17	168.74 (13)	C42—C41—H41A	108.7
V16—O39—V17	83.92 (9)	N6—C41—H41B	108.7
V12—O39—V11	87.92 (9)	C42—C41—H41B	108.7
V15—O39—V11	169.44 (13)	H41A—C41—H41B	107.6
V19—O39—V11	84.85 (9)	C41—C42—H42A	109.5
V16—O39—V11	83.43 (9)	C41—C42—H42B	109.5
V17—O39—V11	83.93 (9)	H42A—C42—H42B	109.5
V15—O40—V12	102.47 (11)	C41—C42—H42C	109.5
V15—O40—V20	100.88 (11)	H42A—C42—H42C	109.5
V12—O40—V20	94.44 (10)	H42B—C42—H42C	109.5
V15—O40—V14	88.50 (10)	C44—C43—N8	112.5 (4)
V12—O40—V14	168.52 (14)	C44—C43—H43A	109.1
V20—O40—V14	86.67 (9)	N8—C43—H43A	109.1
V15—O40—V18	89.66 (10)	C44—C43—H43B	109.1
V12—O40—V18	91.78 (10)	N8—C43—H43B	109.1
V20—O40—V18	166.28 (13)	H43A—C43—H43B	107.8
V14—O40—V18	84.84 (9)	C43—C44—H44A	109.5
V15—O40—V13	168.43 (13)	C43—C44—H44B	109.5
V12—O40—V13	86.56 (9)	H44A—C44—H44B	109.5
V20—O40—V13	85.42 (9)	C43—C44—H44C	109.5
V14—O40—V13	82.14 (9)	H44A—C44—H44C	109.5
V18—O40—V13	82.76 (9)	H44B—C44—H44C	109.5
V17—O41—V11	115.55 (14)	C46—C45—N8	113.3 (4)
V12—O43—V17	108.85 (13)	C46—C45—H45A	108.9
V12—O43—V18	109.07 (13)	N8—C45—H45A	108.9
V17—O43—V18	100.10 (12)	C46—C45—H45B	108.9
V13—O45—V18	116.34 (14)	N8—C45—H45B	108.9
V18—O46—V14	116.53 (14)	H45A—C45—H45B	107.7
V15—O47—V18	107.37 (12)	C45—C46—H46A	109.5
V15—O47—V17	108.97 (13)	C45—C46—H46B	109.5
V18—O47—V17	98.80 (12)	H46A—C46—H46B	109.5
V16—O48—V17	114.74 (14)	C45—C46—H46C	109.5
V19—O49—V11	113.73 (14)	H46A—C46—H46C	109.5
V19—O50—V12	106.19 (12)	H46B—C46—H46C	109.5
V19—O50—V20	108.62 (12)	C48—C47—N8	113.0 (3)
V12—O50—V20	106.87 (12)	C48—C47—H47A	109.0
V20—O51—V13	112.99 (13)	N8—C47—H47A	109.0
V20—O52—V14	113.96 (14)	C48—C47—H47B	109.0
V15—O54—V20	105.19 (11)	N8—C47—H47B	109.0
V15—O54—V19	106.63 (11)	H47A—C47—H47B	107.8
V20—O54—V19	96.22 (10)	C47—C48—H48A	109.5
V15—O54—H54	115.5	C47—C48—H48B	109.5
V20—O54—H54	115.5	H48A—C48—H48B	109.5
V19—O54—H54	115.5	C47—C48—H48C	109.5
V19—O56—V16	113.25 (13)	H48A—C48—H48C	109.5
V19—O56—H56	123.4	H48B—C48—H48C	109.5

### Hydrogen-bond geometry (Å, °)

**Table:**

*D*—H···*A*	*D*—H	H···*A*	*D*···*A*	*D*—H···*A*
N1—H1···O3	0.88	2.06	2.937 (4)	175
N2—H2···O14	0.88	1.89	2.765 (4)	176
N3—H3···O41	0.88	1.82	2.699 (4)	175
N4—H4···O9	0.88	2.13	2.968 (9)	160
N4'—H4'···O9	0.88	1.99	2.830 (16)	158
N5—H5···O26	0.88	2.39	2.981 (5)	125
N5—H5···O28	0.88	2.38	3.187 (5)	153
N6—H6···O51	0.88	1.96	2.823 (4)	167
N7—H7···O34	0.88	2.33	3.061 (4)	141
N7—H7···O45	0.88	2.44	3.156 (5)	139
N7—H7···O46	0.88	2.40	3.139 (4)	142
N8—H8···O52	0.88	1.91	2.766 (4)	162
O15—H15···O18^i^	0.84	1.99	2.819 (4)	171
O17—H17···O16^i^	0.84	1.90	2.715 (4)	162
O54—H54···O55^ii^	0.84	1.88	2.686 (4)	159
O56—H56···O53^ii^	0.84	1.94	2.775 (4)	177

Symmetry codes: (i) −*x*+1, −*y*+1, −*z*; (ii) −*x*+2, −*y*, −*z*+2.
